# A circadian clock regulates efflux by the blood-brain barrier in mice and human cells

**DOI:** 10.1038/s41467-020-20795-9

**Published:** 2021-01-27

**Authors:** Shirley L. Zhang, Nicholas F. Lahens, Zhifeng Yue, Denice M. Arnold, Peter P. Pakstis, Jessica E. Schwarz, Amita Sehgal

**Affiliations:** 1 Chronobiology and Sleep Institute, Perelman School of Medicine at the University of Pennsylvania, Philadelphia, PA USA; 2 Howard Hughes Medical Institute, Perelman School of Medicine at the University of Pennsylvania, Philadelphia, PA USA; 3 Institute for Translational Medicine and Therapeutics (ITMAT), Perelman School of Medicine, University of Pennsylvania, Philadelphia, PA USA

**Keywords:** Circadian rhythms, Blood-brain barrier, Circadian rhythms and sleep

## Abstract

The blood-brain barrier (BBB) is critical for neural function. We report here circadian regulation of the BBB in mammals. Efflux of xenobiotics by the BBB oscillates in mice, with highest levels during the active phase and lowest during the resting phase. This oscillation is abrogated in circadian clock mutants. To elucidate mechanisms of circadian regulation, we profiled the transcriptome of brain endothelial cells; interestingly, we detected limited circadian regulation of transcription, with no evident oscillations in efflux transporters. We recapitulated the cycling of xenobiotic efflux using a human microvascular endothelial cell line to find that the molecular clock drives cycling of intracellular magnesium through transcriptional regulation of *TRPM7*, which appears to contribute to the rhythm in efflux. Our findings suggest that considering circadian regulation may be important when therapeutically targeting efflux transporter substrates to the CNS.

## Abstract (web summary)

The blood-brain barrier is critical for neural function. Here, the authors show that efflux of xenobiotics through the BBB follows a circadian rhythm in mice and human cells.

## Introduction

The blood–brain barrier (BBB) is a structure in the CNS vasculature that insulates the brain from the periphery. A key function of the BBB is to protect the brain from xenobiotics and other potentially harmful environmental insults; however, it also prevents diagnostic and therapeutic drugs from entering the brain^1,2^. Understanding regulation of the BBB is important for addressing effects of the periphery on the brain and also for improving methods of drug delivery. The BBB is comprised of capillary endothelial cells, surrounded by astrocytic endfeet and pericytes^3,4^. BBB endothelial cells constitute a polarized cell layer held together with tight junctions and characterized by low transcytosis, which together restrict paracellular and transcellular passage of molecules into the brain^5,6^. The luminal membrane of the BBB has high concentrations of efflux transporters, notably ABCB1 (ATPase binding cassette also known as permeability glycoprotein [Pgp] and multi-drug resistant protein 1 [MDR1]), which is an ATP-dependent pump with broad substrate specificity^7,8^.

Circadian clocks are endogenous timekeeping processes that cycle with a period of ~24 h and drive rhythms in most physiological processes. A synchronizing central clock resides in the suprachiasmatic nucleus in the hypothalamus; however, the core molecular clock is present in nearly every cell in mammals^9,10^. These peripheral clocks share the same clock machinery as the central clock, but regulate tissue-specific genes and functions^11–14^. The basic mechanism of the clock is a transcription-translation feedback loop in which the Period (*Per*) and Cryptochrome (*Cry*) genes are rhythmically transcribed and the two proteins negatively regulate their transcriptional activators, BMAL1 (brain and muscle aryl hydrocarbon receptor nuclear translocator-like protein 1) and either CLOCK (circadian locomotor output cycles kaput) or NPAS2 (neuronal PAS domain protein)^15^. Because of the pervasiveness of circadian rhythms in cellular processes^16,17^, they are likely involved in the tissue-response to circulating factors, including drugs; however, the extent to which the circadian clock regulates access of molecules/drugs to different mammalian tissues, in particular to the brain, is largely unknown.

To address time-of-day influences on the BBB, we eliminated the molecular clock specifically in the mouse endothelium and used a xenobiotic to measure ABCB1-mediated efflux from the brain. We find that the BBB endothelium has a cell autonomous circadian clock that is required for the generation of rhythms of efflux through ABCB1. We then examined molecular mechanisms of clock-mediated control by conducting transcriptomic analysis using RNA-seq. Surprisingly, unlike the robust rhythms of RNA expression noted in other tissues, very few transcripts cycle in the endothelium and these do not include efflux transporters. We recapitulated efflux cycling in human microvascular endothelial cells and demonstrate oscillations in the level of intracellular free magnesium, a required cofactor for robust efflux. We find binding of BMAL1 to the promoter of the highly expressed magnesium transporter *TRPM7* as well as cycling of *TRPM7* transcript and protein. Together these data suggest a model in which BMAL1 regulates *TRPM7* transcription, which affects intracellular magnesium homeostasis in a rhythmic fashion and produces time-of-day changes in efflux. Our data show the interaction of the molecular clock with BBB function in mammalian cells.

## Results

### Efflux of xenobiotics at the mouse BBB is gated by the circadian clock

**Fig. 1:**
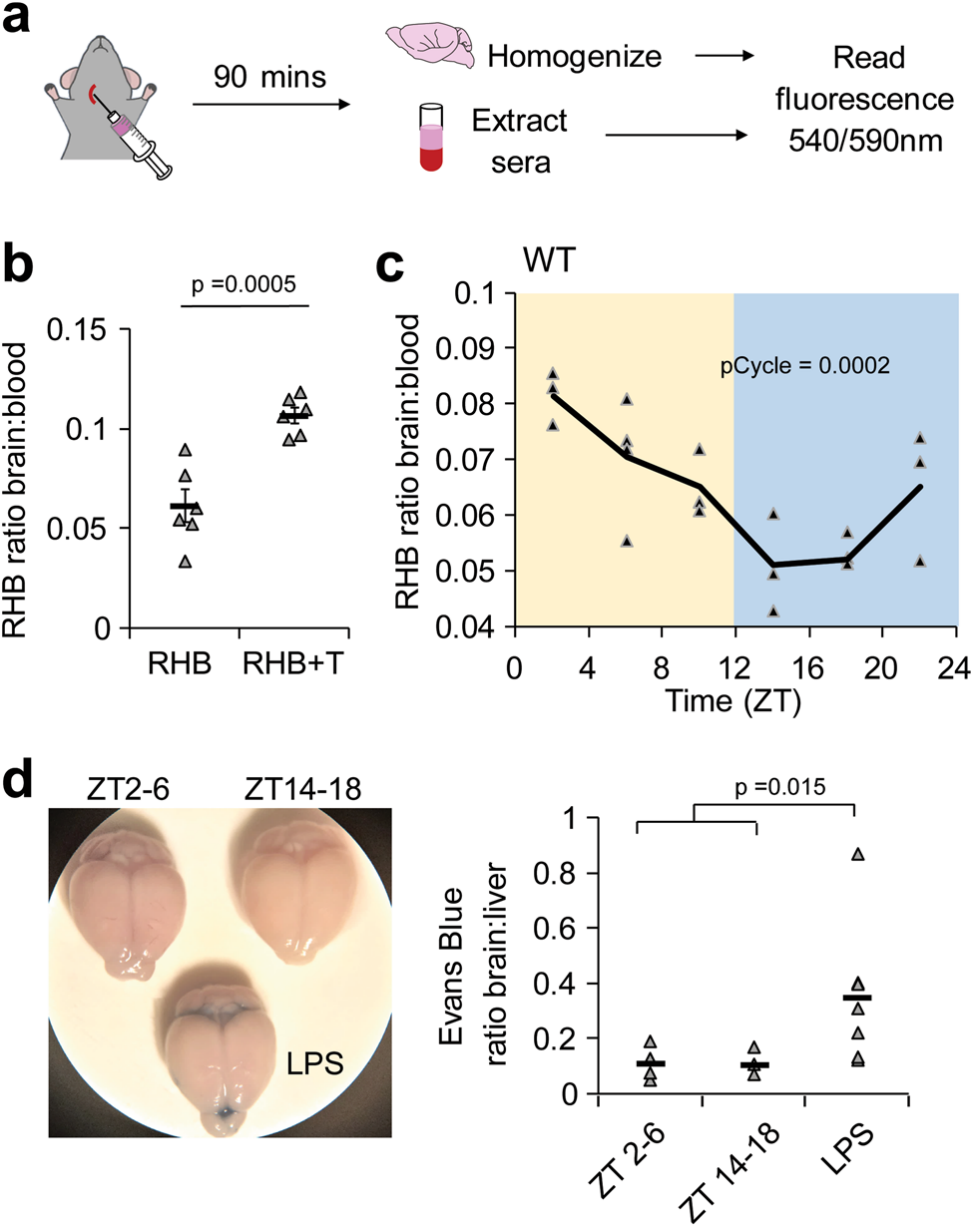
Efflux of ABCB1 substrate from the brain oscillates over the course of the day. **a** Schematic of experiment. The ABCB1 substrate RHB was intravenously injected via jugular vein into mice. Mice were allowed 90 min to recover and brains and sera were collected. Fluorescence was read at ex540/em590nm using a plate reader. **b** Brain RHB levels are regulated by ABCB1-mediated efflux. RHB or RHB and the ABCB1-inhibitor tariquidar was intravenously injected into WT mice. Individual mice are shown with triangle markers and means ± SEM are shown (*n* = 6; 2 independent experiments). **c** RHB injection was performed on WT mice at indicated time points. Individual points are shown with triangle markers and lines represent the mean. pCycle value was calculated by JTKCycle analysis (*n* = 19; 3 independent experiments). **d** Absence of vascular leakage in both day and night. Evans Blue was intravenously injected into mice untreated or treated with LPS 24 h prior. Animals were sacrificed and brains and liver were collected after 30 min. Evans Blue was extracted from tissue and amount was measured at absorbance 620 nm. Individual data points and means are shown. *n* = 8 control, 6 LPS-treated; 2 independent experiments. *p*-value was determined by Student’s T-test.

Activity of efflux transporters at the BBB is an important determinant of BBB permeability^18^. To measure brain efflux, we intravenously injected Rhodamine B (RHB), a small, lipophilic, fluorescent compound that is a known substrate of ABCB1 (p-glycoprotein) into the jugular vein of the mouse. After 90 min, we harvested the blood, sacrificed and perfused the mouse, and harvested the brain. The amount of RHB fluorescence was measured in the serum and brain homogenate (Fig. 1a). To verify that the level of RHB measured in the brain was a result of biological processes rather than experimental error (such as contamination by blood), we used the ABCB1-specific noncompetitive inhibitor tariquidar (*K*_d_ = 5.1 ± 0.9 nM) to block efflux of RHB. We also controlled for possible differences in blood concentrations of RHB, by normalizing RHB levels in the brain to serum levels. We found that administering tariquidar increased the level of RHB in the brain relative to the serum (Fig. 1b), verifying that RHB is effluxed by ABCB1 at the mouse BBB.

To determine whether efflux cycles through the circadian day, we intravenously injected RHB into WT mice at 6 time points throughout the circadian day (zeitgeber [ZT] 2, 6, 10, 14, 18, 22 with lights on and off corresponding to ZT0 and ZT12, respectively) and measured the brain–serum ratio 90 min later. We find that the level of RHB in the brain oscillates throughout the day with the peak early after lights on and trough after lights off, indicating that p-glycoprotein-mediated efflux is highest at night, when the animals are active (Fig. 1c). Levels of RHB in the serum did not show differences over time (Supplementary Fig. 1a).

Higher blood flow due to increased cardiac output and blood pressure during the active period of the mice may increase RHB circulation to the brain^19^; however, in our experiments we find decreased RHB levels in the brain at night, suggesting that increased blood circulation does not explain these results. Nevertheless, to ensure that our measurements reflect efflux over time, we sought to determine the amount of RHB lost from the brain at different times of day. We first measured the RHB brain–serum ratio over the circadian day at 60 min post-injection and found, as expected, that RHB accumulated in the brain to higher levels than at 90 min and the RHB brain–serum ratio trended toward a 24-h cycle (JTK pCycle = 0.1) (Supplementary Fig. 1b). By comparing the RHB blood-serum ratio at 60 and 90 min post-injection, we extrapolated the rate of efflux per minute for each ZT time and found that the highest rate was indeed at night (Supplementary Fig. 1c).

To determine whether gross vascular leakage changes over the day and night, which would contribute to RHB accumulation in the brain, we tracked the level of serum albumin in the brain using the high affinity Evans Blue dye. Wild-type mice were given intravenous injections of Evans Blue in the morning (ZT2-6), night (ZT14-18), or 24 h after treatment with vascular leak-inducing inflammatory stimulus, lipopolysaccharide (LPS), and the brain and liver were assessed 30 min after injection. Half of the mice given LPS exhibited Evans Blue staining in the brain, but uninflamed mice did not exhibit BBB vascular leakage in the morning or night (Fig. 1d), indicating that rhythms of RHB in the brain are not generated by vascular leakage.

**Fig. 2:**
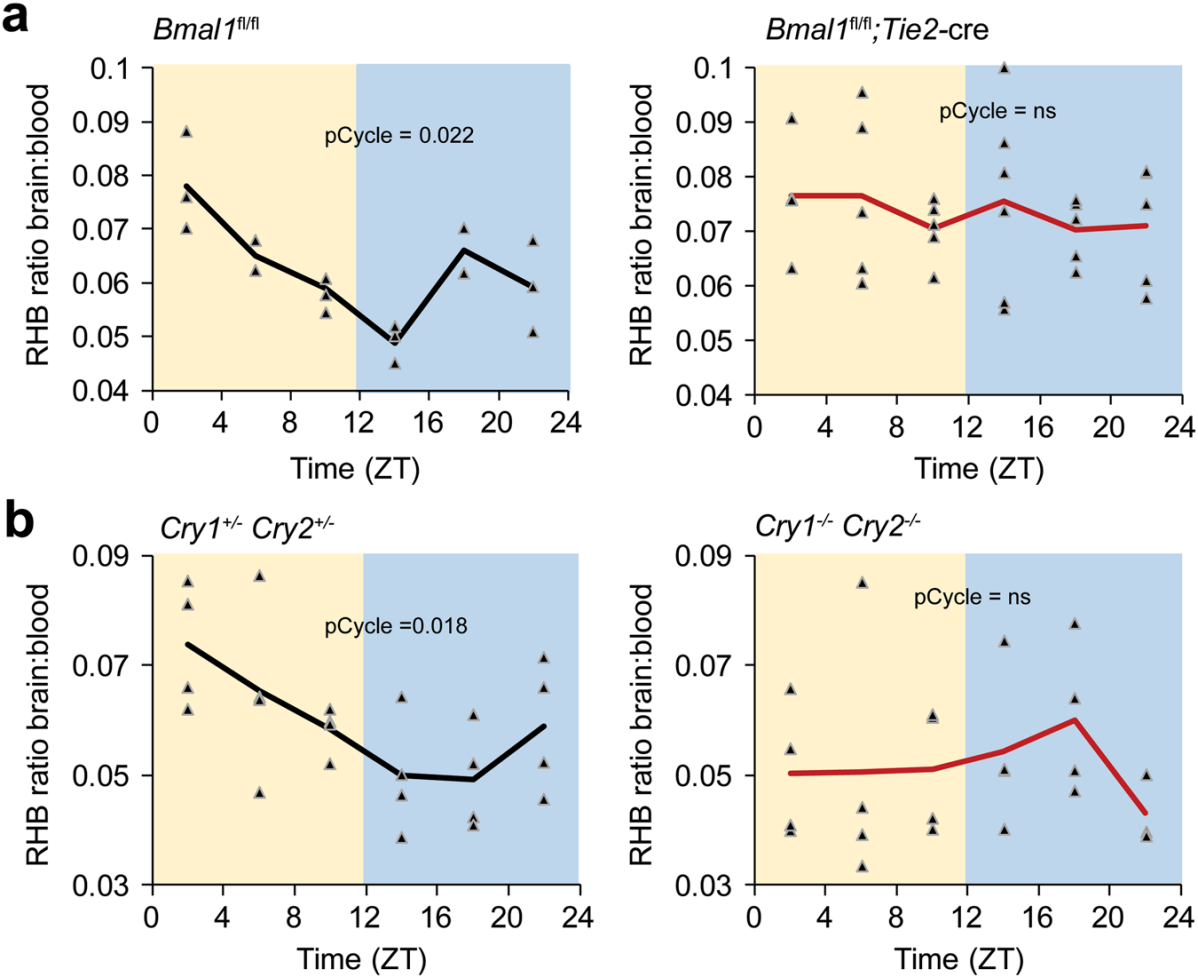
A circadian clock in the BBB regulates brain permeability of RHB. RHB was intravenously injected via jugular vein into mice. Mice were allowed 90 min to recover and brains and sera were collected. Fluorescence was read at ex540/em590nm using a plate reader. **a** Clock ablation in endothelial cells disrupts the brain permeability rhythm to RHB. RHB was injected into control (*n* = 18; 4 independent experiments) or endothelial-specific *Bmal1*-deficient (*n* = 31; 4 independent experiments) mice at indicated time points. **b** Global circadian disruption ablates brain permeability rhythm to RHB in mice. RHB was injected into *Cry1/2* het (*n* = 24; 3 independent experiments) and *Cry1/2* DKO (*n* = 23; 4 independent experiments) mice at indicated time points. Individual mice are shown with triangle markers, and lines represent the mean. pCycle values were calculated by JTKCycle analysis.

To determine whether this oscillation was due to a circadian clock within the blood–brain barrier, we used an endothelial-specific (*Tie2*) cre recombinase to delete a floxed exon of *Bmal1/Arntl* (*Bmal1*^*fl/fl*^), which is required for the generation of circadian rhythms. Control mice (*Bmal1*^*fl/fl*^) have a robust rhythm in RHB efflux; however, in the absence of a functional clock in the BBB endothelium (*Bmal1*^*fl/fl*^; *Tie2*-cre), the rhythms are lost and retention of RHB in the brain is high (Fig. 2a, Supplementary Fig. 2a). We then injected Evans Blue to assay for vascular leak and did not observe any differences between control and mutant mice (Supplementary Fig. 2b). To confirm circadian control, we obtained mice mutant for the Cryptochrome 1, Cryptochrome 2 proteins (*Cry1*^−/−^*Cry2*^−/−^), which repress Bmal1 function. We find that *Cry1*^−/−^*Cry2*^−/−^ also lack RHB efflux rhythms compared to the heterozygous (*Cry1*^+/−^*Cry2*^+/−^) controls (Fig. 2b). Because BMAL1 is part of the positive arm of the circadian clock while CRY1 and CRY2 are part of the negative arm, we expected that deletion of *Bmal1* or *Cry1/2* would have opposing effects on the level of RHB in the brain and indeed *Cry1*^*−/−*^*Cry2*^*−/−*^ mice trended toward lower levels of RHB in the brain (Supplementary Fig. 2c). The high variability of the *Cry1*^*−/−*^*Cry2*^*−/−*^ mice may be due to some paravascular leakage from increased inflammation in the mice^20^. Together, these data suggest that the circadian clock in the BBB regulates RHB efflux from the brain.

### Few circadian transcripts in endothelial cells in the mouse brain

**Fig. 3:**
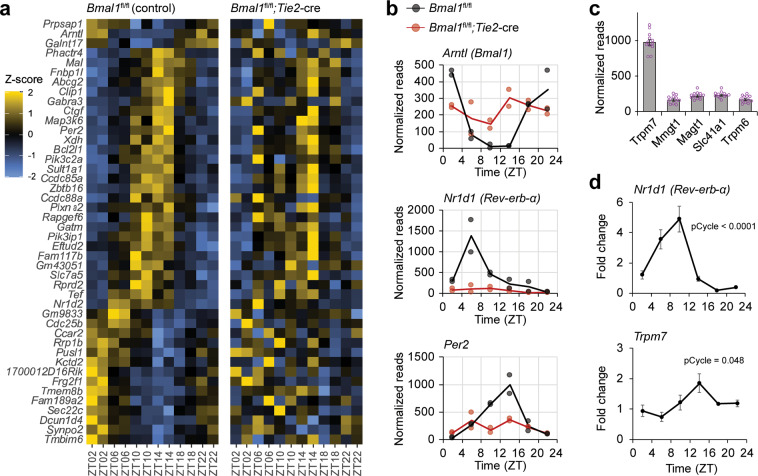
Cycling of circadian clock genes and a highly expressed magnesium transporter in brain endothelial cells. Control or endothelial-cell specific Bmal1-deficient mice were collected at ZT2, ZT6, ZT10, ZT14, ZT18, and ZT22 (*n* = 12; 6 time points, 2 independent experiments); brains were dissected and endothelial cells were isolated by FACS using CD31 antibody. RNA was extracted and sequenced with HiSeq. **a** Few transcripts cycle in BBB endothelial cells. Heatmap of cycling transcripts of control animals (Meta2d, *q* < 0.6) is organized by peak and trough with corresponding transcripts of *Bmal1*-deficient endothelial cells. **b** Expression of core circadian genes shows robust rhythms in mouse BBB. Transcripts of controls (black) are rhythmic (pCycle <0.001) while *Bmal1*-deficient endothelial cells (red) are not. pCycle values were calculated by Meta2d analysis adjusted for multiple comparisons. **c** *Trpm7* is highly expressed in BBB endothelium. Expression of known magnesium transporters in controls (*n* = 12; 2 independent experiments) are shown as means ± SEM. **d** *Trpm7* shows rhythmic expression in brain endothelial cells. qPCR of sorted brain endothelial cells (*n* = 24; 6 time points, 4 independent experiments) were probed for *Nr1d1* and *Trpm7* expression across the circadian day normalized to *GAPDH*. pCycle values were calculated by JTKCycle analysis.

To determine the mechanism of circadian clock regulation, we first profiled the transcriptome of brain endothelial cells at different times of day in *Bmal1*^*fl/fl*^ control and *Bmal1*^*fl/fl*^*; Tie2-cre* mutant mice. We homogenized and processed brains and used fluorescence-activated cell sorting (FACS) to isolate the brain endothelium marked with anti-CD31 (Supplementary Fig. 3a). Based upon previous single-cell sequencing data from brain endothelial cells, we surmise that ~80% of the cells we isolated are from capillaries that comprise the BBB^21,22^. RNA was extracted from the isolated endothelial cells and used to prepare strand-specific libraries with the Clontech Pico V2 kit for 100 bp paired-end sequencing on an Illumina HiSeq 4000. The resulting reads were mapped to the mouse reference genome using STAR^23^, and gene-level normalization and quantification were performed using the Pipeline Of RNA-seq Transformations (PORT). We compared the transcriptomes of the collected brain endothelial cells from control and mutant mice and found comparable levels of claudins and occludins (Supplementary Fig. 3b), indicating that the characteristic signature of BBB-forming endothelial cells in terms of tight junction expression level is unaffected by *Bmal1* loss. Finally, the transcriptome of the brain endothelial cells was analyzed for 24-h rhythmic expression patterns using MetaCycle. Few genes (45 genes with Meta2d *q* < 0.6) were rhythmic in controls and even fewer (6 genes with Meta2d *q* < 0.6) in endothelial-specific *Bmal1* knock-outs (Supplementary Datasets 1–3; Fig. 3a). These data are surprising as transcriptomic analysis of other tissues has revealed cycling of up to 20% of the genome^24^, while here we find that <0.5% of transcripts of the brain endothelial cells are rhythmic. In a previous study of vascular endothelial cells that were isolated and synchronized by serum shock, 229 genes were found to be upregulated by Bmal1/CLOCK, suggesting heterogeneity among endothelia^25^. Isolation of a single cell type might, however, be expected to yield a lower proportion of cycling genes, given that genes expressed rhythmically vary from cell type to cell type and tissue analysis likely represents the sum total of rhythmic genes across several cell types. This is supported by recent single-cell RNA-seq of SCN cells, which revealed cycling of an average of 5% of transcripts in any given cell type^26^, although a higher proportion of the transcriptome cycles in whole tissue SCN^13^. Nonetheless, the number of cycling transcripts we observed is strikingly low given the robustness of the cycling in BBB function (see Figs. 1 and 2). Expression of core clock genes cycled in controls but not in mutants, revealing that the brain endothelium has an autonomous clock (Fig. 3b). Clock output genes also cycled (Supplementary Fig. 3c) in control brain endothelial cells although some were not represented in the 45 cycling genes suggesting that the algorithm used underestimates the number of clock-dependent genes. This could be due to the overall low number of sequence reads and/or heterogeneity of circadian expression within brain endothelial cells.

We did not observe circadian regulation of brain endothelium-specific genes. Several genes which exhibited robust cycling in the controls, such as the amino acid transporter *Slc7a5a*, were also rhythmic in the mutants suggesting that rhythms in other tissues can compensate or that the rhythms are dependent on oscillatory factors like sleep or light. Since ABC transporters are known to regulate xenobiotic efflux, we examined the transcripts of ABC-family transporters and found no consistent phases over time (Supplementary Fig. 4a). *Abcb1a*, the transporter relevant for RHB efflux and most homologous to human *ABCB1* is highly expressed in the BBB, but was not rhythmic or dependent on *Bmal1* (Supplementary Fig. 4b). One ABC efflux transporter, *Abcg2*, was rhythmic by Meta2d, but it was not dependent on *Bmal1* (Supplementary Fig. 4b). Together these results suggest that the circadian clock does not regulate ABC transporters at the level of transcription.

Previous reports have suggested that circadian clocks regulate intracellular magnesium concentrations^27^. Since magnesium is a necessary cofactor in the regulation of ABC transporters, we examined whether its regulation was circadian clock-dependent. We evaluated the RNA-seq results in control animals for all the known magnesium transporters and found *Transient receptor potential cation channel, subfamily M, member 7 (Trpm7)* to be highly expressed (Fig. 3c). *Trpm7* transcripts in control animals trended toward having a rhythm, although this was not statistically significant (Supplementary Fig. 4c). We then sorted BBB from WT mice, extracted RNA, and used real-time PCR to quantify the level of *Trpm7* transcripts, verifying that the mRNA levels are indeed rhythmic (Fig. 3d). This suggested the possibility that the clock regulates intracellular magnesium homeostasis to affect efflux.

### Oscillations of intracellular magnesium levels likely regulate efflux in a cultured human brain endothelial cell (BEC) line

**Fig. 4:**
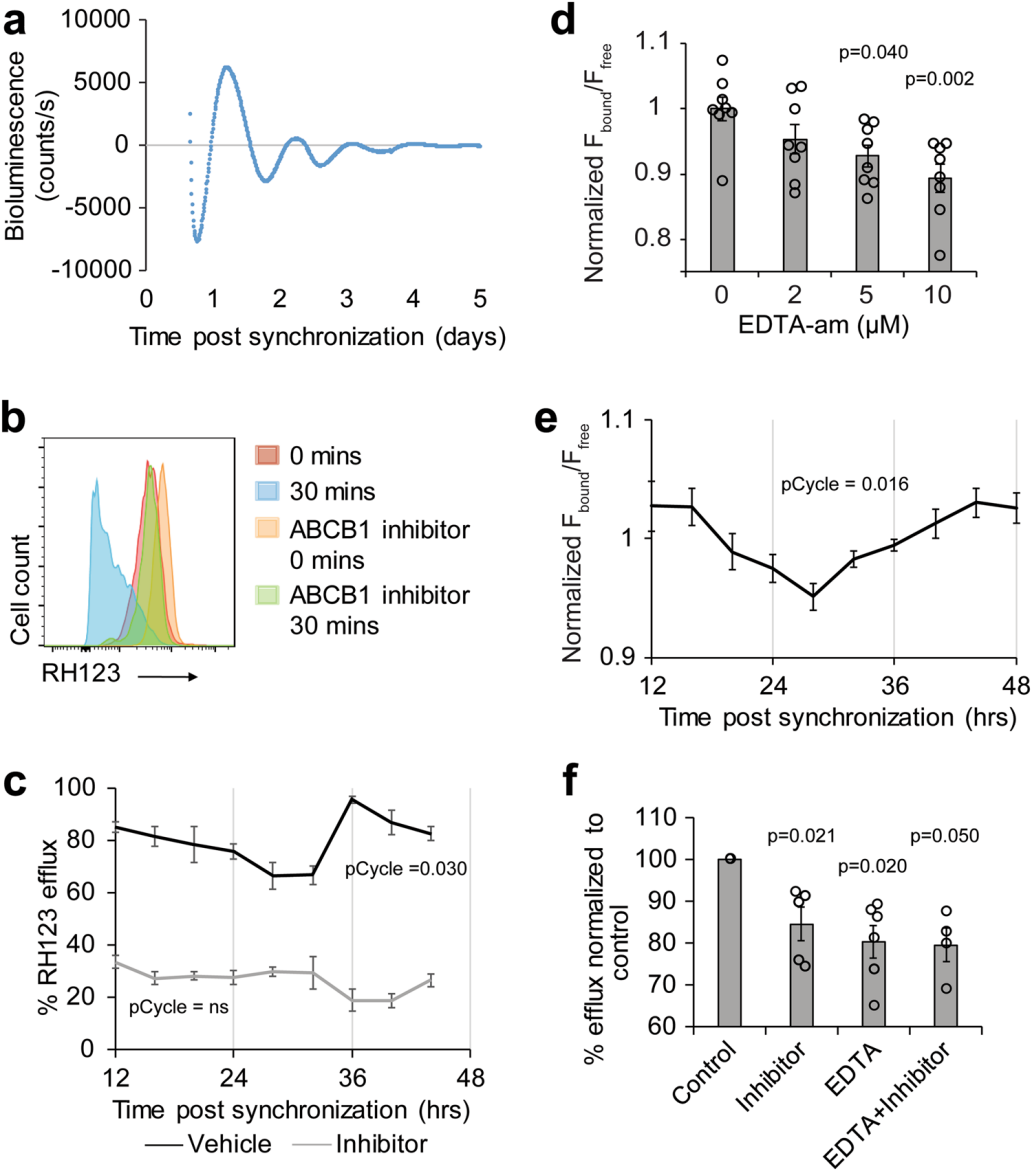
Cyclic efflux in human BECs is likely driven by intracellular magnesium oscillations. **a** Human BEC contain a circadian clock. A stable line was established from hCMEC/D3 cells containing *Per2-dLuc*. Cells were synchronized with a 30 min pulse of dexamethasone and bioluminescent counts were measured over 5 days with a luminometer (LumiCycle 32). Data were analyzed with LumiCycle software and representative detrended plot is shown. **b** Verapamil inhibits RH123 efflux. Suspended cells were incubated with RH123 with or without the ABCB1-inhibitor verapamil on ice for 15 min. Excess RH123 was removed and half of the culture was incubated at 37 °C for 30 min to allow for optimal efflux conditions while the rest remained on ice. The amount of intracellular RH123 was determined by flow cytometry. Representative histograms of each treatment condition are shown. **c** Efflux of RH123 from BBB cells is rhythmic. Cells were synchronized with dexamethasone and efflux assay was performed at the indicated time point. The percentage of RH123 fluorescence effluxed in 30 min comparing the level of fluorescence in cultures with or without 37 °C incubation is shown as means ± SEM. Cells were incubated with vehicle (*n* = 81; 9 time points, 9 experiments) or the ABCB1-inhibitor verapamil (*n* = 45; 9 time points, 5 experiments). pCycle values were calculated by JTKCycle analysis. **d** Chelating magnesium reduces ratio of bound to free MagFura2. hCMEC/D3 cells were incubated with MagFura2-AM and indicated doses of EDTA-AM. MagFura was measured at ex330/em490nm (free) ex369/em490nm (bound). Means of normalized fluorescence of bound Magfura2 (F_bound_) over the fluorescence of free Magfura2 (F_free_) ± SEM are shown. *n* = 4, representative of 2 independent experiments. One-way repeat measures mixed effect analysis with Dunnett’s multiple comparisons test was used to compare each experimental group to control. **e** Intracellular magnesium levels oscillate in phase with efflux cycles. hCMEC/D3 cells were incubated with intracellular magnesium indicator Magfura2-AM at the indicated time points after dexamethasone synchronization and measured at ex330/em490 (free) ex369/em490 (bound) using a plate reader. Means of normalized fluorescence of bound Magfura2 (F_bound_) over the fluorescence of free Magfura2 (F_free_) ± SEM are shown (*n* = 50; 10 time points; 5 independent experiments.). pCycle values were calculated by JTKCycle analysis. **f** Reducing intracellular magnesium inhibits efflux. Verapamil and/or EDTA was added to hCMEC/D3 cells and RH123 was measured by flow cytometry. The percent of RH123 of efflux was normalized to control (*n* = 5–8 from 4 independent experiments). One-way ANOVA was used to compare all experimental groups to control with Dunnett’s multiple comparisons test.

We were unable to measure magnesium levels in the mouse BBB because tissue penetrance of magnesium indicators is poor; thus, we used an immortalized human cerebral microvascular endothelial (hCMEC/D3) cell line^28^. First, we validated that the hCMEC/D3 cell line has a circadian rhythm by transducing the cell line with *PER2-luciferase* and measuring luminescence over several days. We find that the cells have a period of ~23 h (Fig. 4a). To determine whether circadian clocks in the cell lines produce oscillations in efflux, we treated hCMEC/D3 cells with dexamethasone to synchronize their endogenous circadian clocks^29^. Twelve to forty-eight hours after synchronization, cells were incubated with the xenobiotic rhodamine 123 (RH123), a previously validated ABCB1 substrate of hCMEC/D3 cells^30^. Fluorescence retained in the cells after 30 min at 37 ˚C compared to fluorescence retained in cells on ice was measured by flow cytometry. Increased retention of fluorescence by incubation with verapamil indicates that the fluorescence loss reflects ABCB1-mediated efflux of RH123 from the hCMEC/D3 (Fig. 4b). The amount of RH123 efflux by hCMEC/D3 cells oscillates over time and the rhythm is lost when efflux is inhibited with verapamil (Fig. 4c).

To measure the level of intracellular magnesium over time, we synchronized the hCMEC/D3 culture and used a ratiometric intracellular magnesium indicator Magfura2-AM. We verified that the indicator was sensitive to the level of intracellular magnesium in the hCMEC/D3 culture by using EDTA-AM to chelate magnesium (Fig. 4d), and then measured magnesium at different times following dexamethasone synchronization. The level of intracellular magnesium cycles roughly in phase with the cycle of efflux (Fig. 4e). Because the amplitude of the change in magnesium levels was low in this assay, we also stably expressed a genetically encoded magnesium FRET sensor, MARIO (magnesium ratiometric indicator for optical imaging) in hCMEC/D3. We measured the ratio of YFP/CFP in the cells for up to 48 h following synchronization with dexamethasone and found that the level of intracellular magnesium was rhythmic (Supplementary Fig. 5a). Use of a magnesium chelator (EDTA-am) verified that the FRET sensor was sensitive to intracellular magnesium, although the sensitivity was much lower than that of Magfura2-AM (Supplementary Fig. 5b). To determine whether magnesium levels affect efflux, we incubated cells with EDTA-am and/or verapamil and performed a RH123 efflux assay. We found similar reduction of efflux if either drug or both were applied to the cells, suggesting that changing concentrations of intracellular magnesium affect the activity of the ABCB1 transporter (Fig. 4f). Together these results suggest that oscillations of intracellular magnesium levels regulate the cycling of efflux activity.

### Magnesium transporter *TRPM7* is a direct target of the circadian clock

**Fig. 5:**
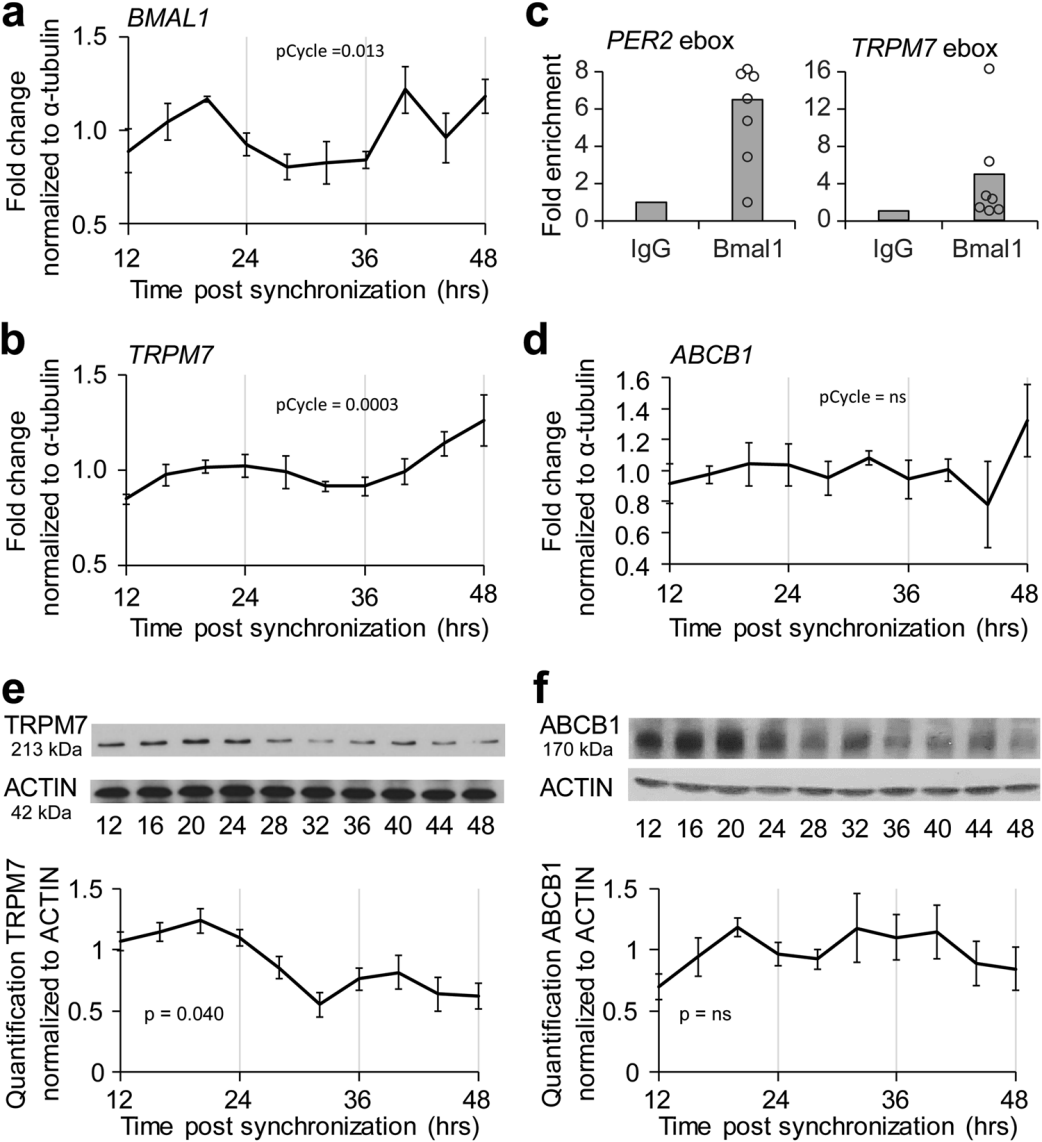
*TRPM7* is a clock-controlled gene in human brain endothelial cell culture. **a**, **b** BMAL1 and TRPM7 cycle in phase following dexamethasone synchronization. hCMEC/D3 mRNA was extracted at indicated time points post-synchronization. Real-time PCR analysis was performed for *BMAL1* and *TRPM7*. Data are shown as means ± SEM (*n* = 40; 10 time points from 4 independent experiments.) pCycle values were calculated by JTKCycle analysis. **c** BMAL1 binds *TRPM7* e-box site. TRPM7 gDNA from hCMEC/D3 cells were immunoprecipitated with either IgG or BMAL1 antibody. Primers for *PER2*, *TRPM7* e-boxes were assessed by real-time PCR. Data are shown as mean fold change of sites binding to BMAL1 normalized to IgG (*n* = 6 plates from 3 independent experiments). **d** mRNA from hCMEC/D3 cultures was extracted at indicated time points post-synchronization. Real-time PCR analysis was performed for *ABCB1*. Data are shown as means ± SEM (*n* = 40 plates; 10 time points; 4 independent experiments). pCycle values were calculated by JTKCycle analysis. **e**, **f** Protein levels of TRPM7, but not ABCB1 oscillate in hCMEC/D3 cultures. Cell lines were synchronized with a pulse of dexamethasone. Cell lysates were collected between 12 and 48 h later. Lysates were blotted with antibodies against TRPM7 or ABCB1 and ACTIN. Representative immunoblot of TRPM7 (*n* = 4; 4 independent experiments) or ABCB1 (*n* = 3; 3 independent experiments) and ACTIN and means ± SEM of quantifications are shown. Quantification of blots was performed in ImageJ. *P*-values were determined using one-way ANOVA to compare among time points.

Our next objective was to understand how the circadian clock affects levels of intracellular magnesium. To determine whether the clock regulates magnesium transporters, we examined transcript levels in hCMEC/D3 cells following dexamethasone synchronization. We found both *BMAL1* and *TRPM7* transcripts cycle relative to tubulin (Fig. 5a, b). Because the acrophase of *BMAL1* preceded the acrophase of *TRPM7*, we assessed whether BMAL1 directly binds the *TRPM7* promoter by using an anti-BMAL1 antibody for chromatin immunoprecipitation (ChIP). Using the Eukaryotic Promoter Database to identify putative BMAL1-binding e-box sites in the *TRPM7* promoter, we then used qPCR to determine the fold enrichment of BMAL1-binding relative to that of IgG controls. The increase in BMAL1 binding to the *TRPM7* e-box is comparable to the level of BMAL1 binding to a known e-box in the *PER2* promoter (Fig. 5c). ChIP-seq data sets have also identified the promoter region of *TRPM7* to be a target of BMAL1^31,32^. By contrast, *ABCB1* transcript remained at comparable levels over a 24-hour period (Fig. 5d) and negligible enrichment of BMAL1 binding was found for two putative e-boxes on *ABCB1* promoter (Supplementary Fig. 6). Together these results are consistent with the idea that *TRPM7*, but not *ABCB1*, is a direct target of the molecular clock.

We then determined whether the transcriptional oscillation of *TRPM7* translates to oscillations in protein levels. Using an antibody against TRPM7 to assess protein expression by immunoblot following dexamethasone synchronization, we found that, indeed, protein levels of TRPM7 oscillate in phase with the mRNA levels (Fig. 5e). Immunoblot of ABCB1 protein shows that levels do not cycle (Fig. 5f), suggesting that the activity of ABCB1 is independent of the level of protein.

**Fig. 6:**
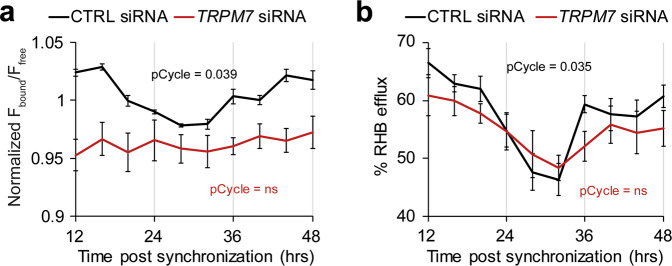
Knockdown of *TRPM7* reduces and dampens rhythms of intracellular Mg^2+^ and xenobiotic efflux. Stable hCMEC/D3 cell lines containing scrambled siRNA and GFP or *TRPM7* siRNA and GFP were made with Lentiviral transduction. Stable lines were synchronized with dexamethasone and assayed at the indicated time points. **a** Knockdown of *TRPM7* results in reduced Mg^2+^ and loss of cycling. Generated cell lines were incubated with intracellular magnesium indicator Magfura2-AM at the indicated time points after dexamethasone synchronization and measured at ex330/em490 (free) ex369/em490 (bound) using a plate reader. Means of normalized fluorescence of bound Magfura2 (F_bound_) over the fluorescence of free Magfura2 (F_free_) ± SEM are shown (*n* = 50; 10 time points, 5 independent experiments). *p* < 0.0001 comparing control to *TRPM7* siRNA cell line by paired T-test, showing less intracellular Mg^2+^ in the *siTRPM7* cell line across time points. pCycle values were calculated by JTKCycle analysis. **b** Knockdown of *TRPM7* reduces xenobiotic efflux and dampens rhythms. Cells were synchronized with dexamethasone and RHB efflux assay was performed at the indicated time point. Control or *TRPM7* siRNA-treated suspended cells were incubated with RHB on ice for 15 min. Excess RHB was removed and half of the culture was incubated at 37 °C for 30 min to allow for optimal efflux conditions while the rest remained on ice. The amount of intracellular RHB was determined by flow cytometry. The percentage of RH123 fluorescence effluxed in 30 min comparing the level of fluorescence in cultures with or without 37 °C incubation is shown as means ± SEM (*n* = 40; 10 time points, 5 independent experiments. pCycle values were calculated by JTKCycle analysis. *p* = 0.0085 comparing control to *TRPM7* siRNA cell line by paired T-test, showing reduced RHB efflux in the *siTRPM7* cell line across time points.

To directly manipulate the level of TRPM7 in the hCMEC/D3 line, we created stable cell lines using lentiviral vectors containing GFP and scrambled siRNA or siRNA targeting *TRPM7*. Interestingly, siRNA knockdown of *TRPM7* resulted in >50% cell loss as well as lower copy numbers (reduced GFP expression), suggesting that TRPM7 is required for survival (Supplementary Fig. 7a). The resultant *siTRPM7* cell line had 4-fold reduction in *TRPM7* transcript levels (Supplementary Fig. 7b), which was sufficient to reduce intercellular free magnesium and abrogate the 24-h oscillation (Fig. 6a). Because of the presence of GFP in our stable cell lines, we could not use RH123 and thus used RHB for ABCB1-dependent efflux experiments. Consistent with the intracellular magnesium levels, the siTRPM7 cell line also showed lower levels and loss of oscillation of RHB efflux compared to the control cell line (Fig. 6b). These results demonstrate that TRPM7 can regulate the levels of intracellular magnesium and ABCB1-mediated efflux.

## Discussion

We report circadian regulation of xenobiotic efflux in both mouse BBB and human cultured brain endothelial cells. The circadian regulation of BBB efflux explains previous findings in which levels of an ABCB1 substrate, quinidine, in the rat brain depended on the timing of administration^33^. The molecular clock in the BBB may also serve to regulate brain levels of endogenous hormones and cytokines through the activity of transporters. Previous reports show concentrations of leptin, interleukin-6, and tumor necrosis factor α have different phases between blood and brain^34–36^. Together, a growing body of work suggests that the BBB is not only physically restrictive, but also temporally restrictive.

A seemingly simple mechanism of clock-control of BBB efflux would be to directly regulate the cycling of transporter transcripts; however, we did not observe a consistent phase among the ABC transporter transcripts. In fact, very few transcripts are clock-controlled genes in the mouse brain endothelium, which is surprising given the extent of circadian regulation of the transcriptome in other tissues^24^ as well as previous reports of cycling ABC-family transporters in the gut^37,38^ and liver^39^. Yet the impact of the clock on cell physiology in the BBB is dramatic. We find the clock regulates intracellular Mg^2+^ levels, which may affect cell physiology more broadly than transcriptional changes. For instance, changing Mg^2+^ or translational machinery may affect the activity or levels of many proteins; however, to achieve the same effect at the transcriptional level, clock proteins would need to bind and regulate each individual transcript.

Our results suggest that the efficiency of xenobiotic efflux of the BBB is regulated, at least in part, by oscillating free magnesium levels. This is consistent with previous reports of oscillating free magnesium concentrations and magnesium transporters in other cell types^27,40^. Because Mg^2+^ is a cofactor for hundreds of MgATP-dependent enzymes, even modest changes in cytosolic Mg^2+^ may serve to regulate cellular energy expenditure over the course of a day^41^. Indeed, we find that inhibition of intracellular Mg^2+^ in the range of the daily variation can reduce efflux. In the BBB, such changes in intracellular free magnesium may also regulate the activity of other BBB transporters, such as those for amino acids and glucose transporters, if Mg^2+^ is rate-limiting for transport. In addition to magnesium transporter, changes in intracellular calcium may contribute to oscillations of Mg^2+^.

**Fig. 7:**
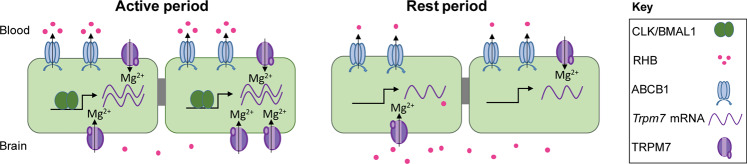
Model of clock regulation of xenobiotic efflux at the BBB. Core circadian clock transcription factors BMAL1 and CLOCK activate transcription of *Trpm7*. Increased TRPM7 allows for higher intracellular free magnesium, which regulate the activity of xenobiotic transporters. Higher transporter activity decreases the level of xenobiotics in the brain. In the absence of BMAL1/CLOCK, TRPM7 and therefore free intracellular magnesium is low. Lower levels of magnesium are associated with decrease the activity of the xenobiotic transporters, thus xenobiotics are retained in the brain.

Together, our data suggest a model in which BMAL1 in the BBB endothelium drives transcript and protein expression of TRPM7 during active periods (Fig. 7). The increase in TRPM7 channel likely results in higher concentrations of intracellular free magnesium, which increases the activity of the ABCB1 transporter and thereby the level of xenobiotic efflux, reducing the amount of RHB in the brain. During resting periods, BMAL1 is low, reducing the level of TRPM7 and subsequently reducing the level of free magnesium in the BBB. Lower levels of intracellular magnesium decrease xenobiotic efflux, resulting in greater brain retention of RHB. In support of this model, loss of the CRY proteins, which are negative regulators of BMAL1, results in lower retention of RHB. However, we cannot exclude the possibility that other factors, such as the recently proposed regulation of the BBB by neuronal activity, contribute to rhythms in efflux^42^.

Our results show that accumulation of xenobiotics in the brain following intravenous injection of mice occurs with an inverted cycle relative to that of a previously described efflux cycle in *Drosophila*^43^. This aligns with the rest:activity rhythms of the organism with highest efflux during the active period and lowest efflux during the resting period. High levels of efflux would be most advantageous during periods of activity because of the presumed increase in exposure to toxins from foraging or injury. Despite the evolutionary conservation of circadian regulation of xenobiotic efflux, we find differences in mechanism between flies and mammals. In flies, the circadian clock uses a non-cell autonomous mechanism to regulate efflux activity of the BBB; however, in mammals the endothelial BBB-clock drives cycling of efflux within the same cell type. Interestingly, though, both systems include an important role for magnesium. In flies, rhythmic expression of gap junctions restricts the passage of magnesium between two layers of the BBB to specific times of day; at these times, magnesium is depleted from the subperineurial layer, which contains efflux transporters and normally has very high magnesium, thereby resulting in a rhythm of magnesium^43^. In mammals, on the other hand, our data suggest that magnesium rhythms are driven by cell-autonomous clock control of a magnesium transporter.

The cycling of ABCB1 activity may have implications in disease settings; for instance in Alzheimer’s disease, where ABCB1 contributes to the clearance of amyloid beta from extracellular spaces of the brain^44,45^. Typically, amyloid beta is either broken down in the brain or cleared through the BBB endothelium or the cerebrospinal fluid. Interestingly, amyloid beta clearance is higher during sleep^46^, whereas here we show the peak activity of ABCB1 during active hours. However, efficient clearance of amyloid beta through the endothelium requires both ABCB1 and LRP1 for phosphatidylinositol binding clathrin assembly protein (PICALM)-mediated transcytosis through BBB endothelium^47^. Despite lower activity of ABCB1, it is possible that transcytosis is higher during sleep, as shown for endocytosis in the Drosophila BBB^48^. Thus, increasing ABCB1 activity at this time could facilitate amyloid beta clearance.

A therapeutic application of this work is the possibility of improving drug delivery to the central nervous system (CNS). Given that many drug targets are clock-controlled genes, cells and tissues have different drug sensitivities throughout the circadian day^49^. Utilizing circadian timing in medicine, also known as chronotherapy or chronomedicine, can improve outcomes and decrease side effects due to lower required doses to achieve the same efficacy^50^. Here, we show that ABCB1 substrates are subject to circadian regulation at the BBB, indicating an additional level of circadian control for many drugs targeted to the CNS; however, other mechanisms of oscillatory regulation could also influence drug concentrations in the brain. Sleep has been shown to increase removal of metabolites from the brain^46^, and robust circadian rhythms in the choroid plexus increase cerebrospinal fluid (CSF) production and turnover during the resting period^51,52^. These mechanisms may allow brain retention of other classes of drugs in a time-of-day-dependent matter, which could inform decisions on optimal chronotherapy. Our results suggest that optimizing circadian timing of drug delivery in regards to BBB permissiveness can achieve increased CNS drug efficacy and should be taken into consideration in developing therapeutic regimens.

## Methods

### Animal care and husbandry

C57BL/6J (#000664), *Bmal1*^*fl/fl*^ (B6.129S4(Cg)-*Arntl*^*tm1Weit*^/J #007668)^53^, and *Tie2cre* (B6.Cg-Tg(*Tek-cre)1Ywa*/J, #008863)^54^ mice were purchased from Jackson Laboratory animal facility. *Cry1,Cry2* double-deficient mice were a gift from Katja Lamia originally from Aziz Sancar^55^. Animals were fed ad libitum and entrained to 12 h:12 h light:dark cycles and given at least 1 day per hour of phase shift prior to experiments. All live animal experiments were performed according to protocols approved by the Institutional Animal Care and Use Committee of the University of Pennsylvania (Philadelphia, PA) in accordance with guidelines set by the NIH (Protocol #806387). Male and female mice were used for gene expression data (RNAseq and qPCR). Only male mice were used in functional experiments due to the decreased risk for surgical failures with their larger size. Mice from the same litters were randomly distributed to environmental cabinets for entrainment for different circadian time points.

### RHB permeability assay in live mice

Mice between 8 and 12 weeks were used for permeability experiments. The surgeon was blinded to the genotype and circadian time of the mice. Mice were anesthesized with continuous isoflurane to maintain surgical plane and given meloxicam (5 mg/kg) and bupivacaine (0.1 mg/kg) subcutaneously. RHB (Sigma) was dissolved in 10% water and 90% PBS and was injected (5 mg/kg) into the jugular vein. Tariquidar (Selleck Chemicals) was suspended in 30% Propylene glycol and 5% Tween 80 and 65% of 5% dextrose in water and coinjected (30 mg/kg) into the jugular vein. Animals that did not receive a proper injection were sacrificed and excluded from analysis (~5–10% of the animals). The mice were allowed 90 min to recover prior to tissue harvest. Animals were anesthetized with isoflurane and blood was collected by retro-orbital bleed and serum was extracted after <10 min of coagulation. Mice were then sacrificed by cervical dislocation and remaining blood was removed through cardiac perfusion of 1x PBS before brains were harvested. Brains were sliced into halves and homogenized in equal volume by weight PBS using a steel ball and TissueLyser II (Qiagen) set to 25 Hz for 2 min. RHB fluorescence was measured with Victor3V Plate Reader (Perkin Elmer) at Excitation/Emission ex540/em590nm. Amount of RHB was determined by standard curve and ratios of brain to blood levels were calculated for each animal.

### Vascular leakage assay

Evans Blue vascular leakage assay was adapted from previous studies^56^. Briefly, mice were anethesized with isoflurance and administered 50 mg/kg Evans Blue via retro-orbital injection. After 30 min, mice were sacrificed and perfused with 10 units/mL heparin in PBS. Brain and liver were harvested and Evans Blue was extracted with formamide and the absorbance of the supernatant was measured at 620 nm using a spectrophotometer (Cytation 5, BioTek). Concentration of Evans Blue was determined using standard curve. For induction of vascular leak, 3 mg/kg LPS was administered to the mice by intraperitoneal injection 24-h prior to injection of Evans blue.

### Isolation of BBB endothelium

Brains were harvested from mice at time points around the circadian day (ZT2, 6, 10, 14, 18, 22). Brains were finely minced in RPMI media containing 10% CCS, incubated at 37 °C with 2 mg/mL collagenase IV (Gibco) and 200 μg/mL DNase I (Roche) for 30 min with shaking at 250 rpm, pipetting up and down once during incubation. Brain homogenate was demyelinated using myelin removal beads II (Miltenyi Biotec) as instructed by the manufacturer’s protocol. Cell suspensions were filtered using a 100 μm cell strainer. Cells were labeled with anti-CD90 (clone M5/49.4.1, BioXell) for 15 min on ice and incubated with BioMag Goat Anti-Rat IgG (Qiagen) for 30 min with rotation, and magnetically separated (Easy Sep). Supernatant was removed and spun down at 600 × *g*. Cells were resuspended in 250 μl, labeled with fluorescence-conjugated anti-CD31 (390, BioLegend), anti-CD90 (53-2.1, BioLegend) antibodies sorted using either a BD FACSAria or BD FACSMelody (BD Biosciences). Dead cells were excluded through 4′,6-diamidino-2-phenylindole uptake. Doublets were excluded through FSC-H by FSC-W and SSC-H by SSC-W parameters. Between 10,000 and 20,000 cells were sorted from a single mouse at >95% purity verified by post-sort analysis. Processing of tissue and BBB isolation takes ~4 h. Data from sort were analyzed using FlowJo V10.6 software (TreeStar).

### RNA sequencing and analysis

RNA was extracted from sorted cells using RNeasy Plus Micro Kit (Qiagen) following the manufacturer’s protocol. RNA quality was assessed using 2100 Bioanalyzer Instrument (Agilent). RNA integrity numbers (RIN) of 6–8 were used to construct libraries. Strand-specific libraries were constructed using the SMARTer Stranded Total RNA-Seq Kit v2 - Pico Input Mammalian (Clontech) and sequenced across three lanes of a HiSeq 4000 (Illumina), generating 150 bp paired-end reads. All of the following analyses used the GRCm38 build of the mouse reference genome and gene models from Ensembl v91, both downloaded from the Ensembl portal^57^. Reads were aligned to the reference genome using STAR v2.5.3a^23^ with the following command line options: --outSAMtype BAM Unsorted; --outSAMunmapped Within KeepPairs; --outFilterMismatchNmax 33; --seedSearchStartLmax 33; --alignSJoverhangMin 8. In addition, STAR was provided with the gene models in GTF format during alignments and the “—outSAMattrRGline” was used to add a unique read group to each of the resulting BAM files (required to merge files below). Note that the three pairs of FASTQ files per library (two for each sequencing lane) were aligned separately. Following alignment, the three BAM files for each library were combined using the SAMtools v1.3.1^58^ merge command with the “-n” argument, which maintains the BAM files in “unsorted” order (i.e., sorted by read ID). The Pipeline Of RNA-seq Transformations (PORT) v0.8.5-beta (https://github.com/itmat/Normalization) was used to perform gene-level normalization and quantification of the aligned data. Cycling transcripts with 24 h rhythmicity were identified using the meta2d function from the MetaCycle v1.1.0R package^59^. The meta2d function was run with the following arguments: outIntegration = “both”, adjustPhase = “predictedPer”, combinePvalue = “fisher”, weightedPerPha = FALSE, cycMethod = c(“JTK”, “LS”), analysisStrategy = “selfUSE”, minper = 24, maxper = 24. For the MetaCycle analyses, we applied a minimum expression filter to remove unexpressed genes. For a gene to be included, it must have a normalized read count of five or greater in at least control six samples, or six *Bmal1* mutant samples.

### hCMEC/D3 cell cultures

Immortalized human brain capillary endothelial cells (Millipore SCC066) were cultured in EndoGRO-MV Complete media kit (SCME004) as recommended by the manufacturer. Cells were tested every 3–5 passages for mycoplasma contamination by DNA Hoechst stain. Cultureware was coated with rat tail collagen type I solution at a concentration of 0.1 mg/mL and was incubated for >1 h at 25 °C. Cells were cultured in an incubator at 37 °C with 5% CO_2_. For synchronization of culture, cells were grown to ~60% confluence and incubated in 200 nM dexamethasone for 30 min. Circadian time points were obtained by staggering the synchronization of different cultures and assaying them at the same time.

### Generation of stable cell lines

#### hCMEC/D3 expressing Per2-dLuc-GFP

Stable hCMEC/D3 cell lines expressing Per2-dLuc-eGFP were generated via lentiviral vector transduction as previously described^60^. Briefly, LentiX 293T cells (Clontech) were grown to 70% confluence in 10 cm dishes, and co-transfected with p*Per2-dLuc-eGFP*, together with the packaging plasmids (pDVPR8.1 and pVSV-G, Addgene) in a 10:1:0.5 ratio, using Lipofectamine 3000 PLUS (Life Tech) according to the manufacturer’s directions. Twenty-four hours following transduction, the >50% transfection efficiency was confirmed via fluorescent microscopy to assess GFP positivity, and the media was exchanged. Forty-eight hours following transduction, the supernatant was collected from the transfected 293T cells, and the supernatant was centrifuged at 1000 × *g* for 5 min to pellet any 293T cells. Finally, stable hCMEC/D3 cell lines were generated by infecting 70% confluent cultures with the recombinant lentiviral vectors with 10 mg/mL polybrene (Sigma-Aldrich) twice over 2 consecutive days, and then individual GFP+ cells were sorted on the FACSMelody (BD Biosciences).

#### hCMEC/D3 expressing siRNA against *TRPM7*

Stable hCMEC/D3 cell lines expressing either scrambled siRNA (control) or *TRPM7* siRNA with GFP were generated via lentiviral vector transduction using commercially available lentiviruses (LVP015-G and iV026186, abm). GFP+ cells were sorted in bulk on the FACSMelody (BD Biosciences). Due to the amount of cell death (50%) visually observed in the *TRPM7* siRNA infected cells, we opted to group the cells to avoid mutations that may be present in individual clones.

### Lumicycle assay

Cells containing *Per2-dLuc-GFP* were seeded in 35 mm dishes at 80% confluence. A 30 min 100 nM dexamethasone (Sigma) pulse was used to synchronize the cells. Real-time bioluminescence of the cells were monitored in LumiCycle recording media (hCMEC/D3 media with 10% FBS, 25 mM Hepes, 4.2 mM sodium bicarbonate pH 7.0) with 200 µM beetle luciferin potassium salt using LumiCycle luminometer (Actimetrics) as previously described^60^. Luminescence data were analyzed using LumiCycle software (Actimetrics).

### Cell culture efflux assay

For measuring efflux of hCMEC/D3 cells, an assay was adapted from Löscher Lab^61^. Cells were cultured and synchronized as described above. Cells were preloaded with 10 µM RH123 (Sigma-Aldrich) in Opti-MEM (Invitrogen) for 15 min on ice. Cells were washed twice to remove excess RH123 and resuspended in Opti-MEM without RH123. Half of the cells were incubated at 37 °C with 5% CO_2_ and the decay of intracellular florescence was measured after 30 min. To control for the amount of RH123 preloaded, half of the culture was harvested and remained on ice. Fluorescence was measured using a BD FACSCanto II (BD Biosciences). For inhibition of efflux, 200 µM verapamil was added to the cells during the 15 min of RH123 preloading and the 30 min of incubation. For inhibition of magnesium, 10 µM of EDTA-am (Setareh Biotech) was added to the cells for the duration of 37 °C incubation.

### Measuring magnesium in cell culture

#### Indicator (MagFura2)

Cells were plated in a 96-well plate 2–3 days prior to the assay. hCMEC/D3 media was removed and cells were incubated in 150 μl Opti-MEM for 10 min (Gibco). Magfura2-AM was suspended in DMSO to make a 5 mM stock solution. A dispersion solution was made by adding 4 μl stock solution and 5 μl of 20% (w/v) Pluronic F-127 (Life Tech) to 1 mL Opti-MEM. Fifty microliters of dispersion solution was added to the culture for 30 min at 37 °C. Cells were washed 2× with PBS and incubated in Opti-MEM for 30 min. Magfura2 was measured at ex330/em490nm and ex369/em490nm using a Cytation plate reader.

#### Genetic sensor (MARIO)

A plasmid encoding a magnesium FRET sensor, MARIO (magnesium ratiometric indicator for optical imaging) was obtained from Dr. Takeharu Nagai^62^. MARIO was cloned into pBABE retroviral vector using BamHI/EcoRI. pBABE-MARIO was transfected into ΦNIX cells using Lipofectamine 3000 PLUS (Life Tech). Twenty-four hours following transfection, the media was replaced. Forty-eight hours following transfection, the supernatant was collected and applied with 10 mg/mL polybrene to a culture of hCMEC/D3. Culture was sorted for YFP+ cells using a FACSMelody (BD Biosciences). The ratio of YFP (ex440/em528nm) and CFP (ex440/em480nm) were measured with a fluorescent plate reader.

### Chromatin immunoprecipitation

Chromatin immunoprecipitation of BMAL1 protocol was adapted from previous report^63^. 1 × 10^6^ cells were fixed with 1% formalin for 10 min. 125 mM glycine was added to stop the cross-linking reaction. Cells were washed with PBS and collected by scraping. Cell pellet was resuspended in swelling buffer (5 mM PIPES pH 8.0, 85 mM KCl, 1% NP40, and protease inhibitor cocktail), homogenized, and collected by centrifugation. Nuclei were resuspended in nuclear lysis buffer (50 mM Tris-HCl pH 8.0, 10 mM EDTA, 1% SDS, and protease inhibitor cocktail) and sonicated to an average length of about 300–500 base pairs, which was confirmed with agarose gel electrophoresis. Samples were diluted 10-fold with IP dilution buffer (16.7 mM Tris-HCl pH 8.0, 0.01% SDS, 1.1% Triton X-100, 1.2 mM EDTA, 167 mM NaCl, and protease inhibitor cocktail) and incubated with anti-BMAL1 antibody (5 μg, ab3350, Abcam) or IgG control antibody overnight at 4 °C. DNA complexes were collected on Dynabeads Protein A and serial washed with dialysis buffer (50 mM Tris-HCl pH 8.0, 2 mM EDTA, and 0.2% sarkosyl) and IP wash buffer (100 mM Tris-HCl pH 9.0, 500 mM LiCl, 1% NP40, 1% Deoxycholate, and protease inhibitor cocktail). Samples were removed from beads using elution buffer (50 mM NaHCO_3_ and 1% SDS). Cross-linking was reversed by overnight incubation with 0.2 M NaCl at 67 °C and samples were then RNase A treated and purified. Real-time PCR was performed using *PER2* and *ABCB1* primers. Percentage of input was calculated and normalized to IgG.

### Real-time PCR

RNA was extracted using RNeasy mini kit (Qiagen) and reverse transcribed to cDNA using random hexamers and Superscript II (Invitrogen). Real-time polymerase chain reaction (PCR) was performed using Sybr Green PCR Master Mix (Applied Biosystems) with the oligonucleotides described in Supplementary Table 4. Assays were run on ViiA7 Real-Time PCR system (Applied Biosystems). Relative gene expression was calculated using the ΔΔCt method normalizing to tubulin.

### Immunoblot

hCMEC/D3 cells were harvested at the indicated time points following dexamethasone synchronization, and lysed with 1x Passive Lysis Buffer (Promega) on ice. Lysates were boiled for 10 min and run on 4–12% gradient Tris-Glycine gels (Life Technologies), transferred to PVDF membranes, and probed with the following antibodies: anti-PGP (1:1000, MDR-1 c219, ThermoFisher), anti-TRPM7 (1:500, EPR4582, Abcam), anti-BMAL1 (1:1000, A302-616A, Bethyl Laboratories), and anti-beta-Actin (1:20,000, mAbcam8224, Abcam). Blots were digitalized with a photo scanner (Canon) and bands were quantified using ImageJ software.

### Statistical analysis

Circadian statistical analysis was performed in R using JTK_CYCLEv3.1^64^. Sample size estimates for mouse experiments were based on previous circadian studies; no fewer than *n* = 12 at 6 time points per 24 h. Variance of data for each time point was similar. Student’s T-tests and ANOVAs were performed in Excel (Microsoft) and/or Prism (GraphPad). Determination of sample size and statistical power was performed with powerandsamplesize.com.

### Reporting summary

Further information on research design is available in the Nature Research Reporting Summary linked to this article.

## Supplementary information

Supplementary Information

Supplementary Data 1

Supplementary Data 2

Supplementary Data 3

Description of Additional Supplementary Files

Reporting Summary

## Data Availability

The data that support the findings of this study are available upon request. Source data are available for Figs. 1b–d, 2, 4c–f, 5, 6. The RNA-sequencing datasets have been deposited in GEO database under accession code GSE135874.

## Source data

Source Data
