# Supplementary material for: A circadian clock regulates efflux by the blood-brain barrier in mice and human cells
Source: Nat Commun. 2021 Jan 27;12:617. doi: 10.1038/s41467-020-20795-9 (PMC7841146; doi:10.1038/s41467-020-20795-9)
Supplement: Supplementary file 1 — Supplementary Information [file 41467_2020_20795_MOESM1_ESM.pdf]

Supplemental Figures

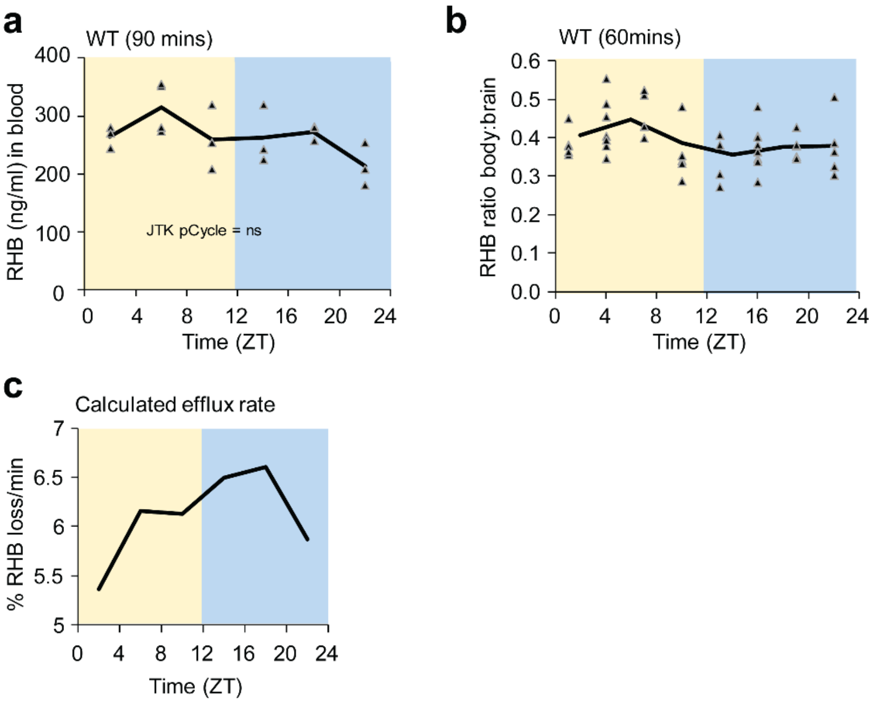

Supplementary Figure 1. Cyclic brain retention of RHB is not affected by levels in the blood. a) Comparable levels of RHB remained in the blood of RHB injected mice thoughout the circadian day. RHB was intravenously injected via jugular vein into mice. Mice were allowed 90 mins to recover and brains and sera were collected. Fluorescence was read at ex/em 540nm/590nm using a plate reader. Brain:blood ratios are shown as mean (line) and individual points (triangles). pCycle values were calculated by JTKCycle analysis. (n=19 from 3 independent experiments) **b**) Brains of RHB injected mice contained much higher levels of RHB at 60min post-injection. RHB was intravenously injected via jugular vein into mice (n=42 from 4 independent experiments). Mice were allowed 60 mins to recover and brains and sera were collected. Fluorescence was read at ex/em 540nm/590nm using a plate reader. Brain:blood ratios are shown as mean (line) and individual points (triangles). **c)** Rate of RHB efflux is higher in the early night. The efflux rate of RHB from the brain was calculated from mean RHB ratios at 60 and 90 mins post injection using the equation [rate = $\ln(B_{60}/B_{90})/t$] where B is the ratio of RHB retained in the brain, t is minutes between the two points.

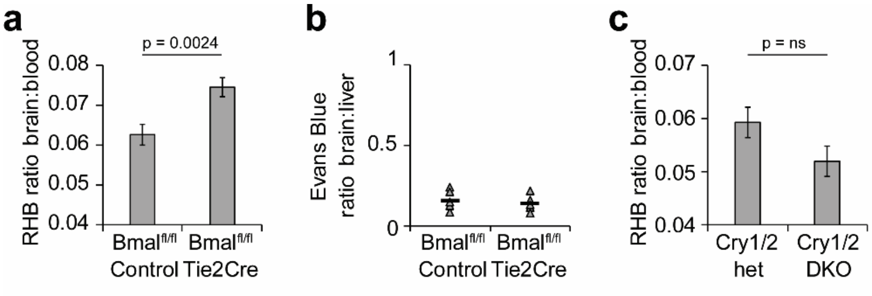

Supplementary Figure 2. Loss of circadian activator (*Bmal1*) results in increased RHB retention. a) Brain RHB retention is higher in mice with clock-deficient blood-brain barrier. RHB was intravenously injected into control (n=18; 4 independent experiments) or endothelial cell specific *Bmal1*-deficient (n=31; 4 independent experiments) mice and brain and serum was collected 90 mins later. Fluorescence was read at ex/em 540nm/590nm using a plate reader. Brain-to-blood ratio is shown as means ± SEM. Individual data points are shown in Fig 2. p-value was determined by Student's T-Test. **b**) Comparable levels of vascular leakage between control and endothelial cell specific *Bmal1*-deficient mice. Evans Blue was intravenously injected into mice between ZT12-16. After 30 mins, brains and liver were collected. Evans Blue was extracted from tissue and amount was measured at absorbance 620nm. Bars represent means ± SEM (n = 5). **c**) Cry1/2 DKO mice trend toward lower levels of RHB retention in the brain. RHB was intravenously injected into Cry1/2 het (n=24; 4 independent experiments) and Cry1/2 DKO mice (n=22; 4 independent experiments). Brain and serum were collected and fluorescence was read at ex/em 540nm/590nm. All time points for the brain-to-blood ratio in Cry1/2 het and Cry1/2 DKO pooled are shown as means ± SEM. Individual data points are shown in Fig 2. p-value was determined by Student's T-Test.

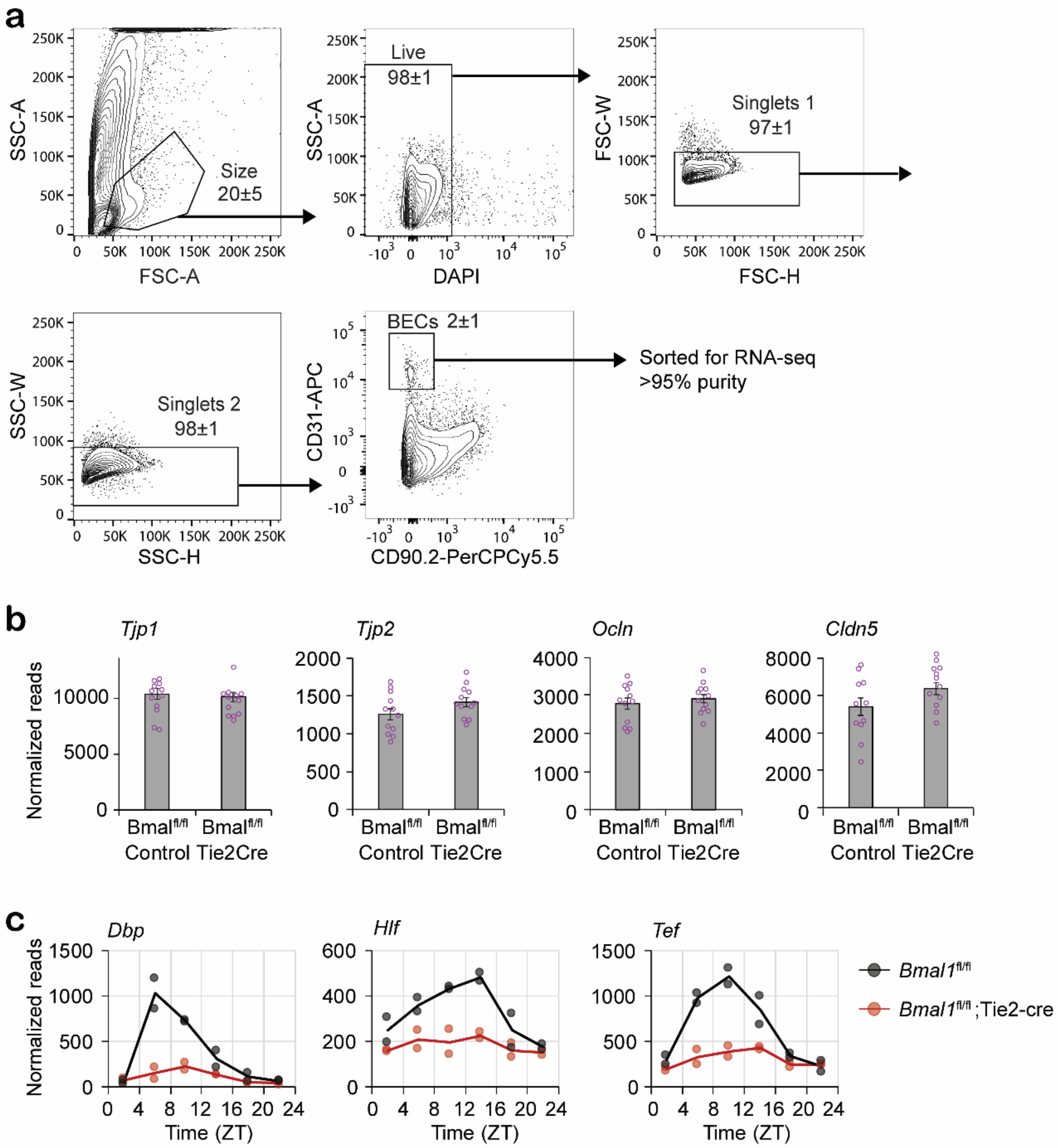

Supplementary Figure 3. Characteristics of brain endothelial cells are comparable between control and *Bmal1*-deficient cells. Brain endothelial cells from control and endothelial cell specific *Bmal1*-deficient were sorted and analyzed by RNAseq (n=12; 2 experiments). **a)** Gating strategy for BECs. Whole brains were dissected, dissociated, and enriched (see methods). RNA was isolated from sorted cells for RNA-sequencing, resulting in data corresponding to Fig 3. Representative plots from one animal are shown with gates and population percentages ± SD. **b)** No change in tight junction genes in the absence of *Bmal1*. Genes involved in the formation of BBB tight junctions were analyzed and pooled across all time points. Data are shown as means ± SEM. **c)** Circadian output genes cycle in brain endothelial cells. Individual plots of clock output genes, *Dbp*, *Hif*, and *Tef*, show rhythms in controls (pCycle <0.05 by Meta2d) but not in *Bmal1*-deficient endothelial cells**.**

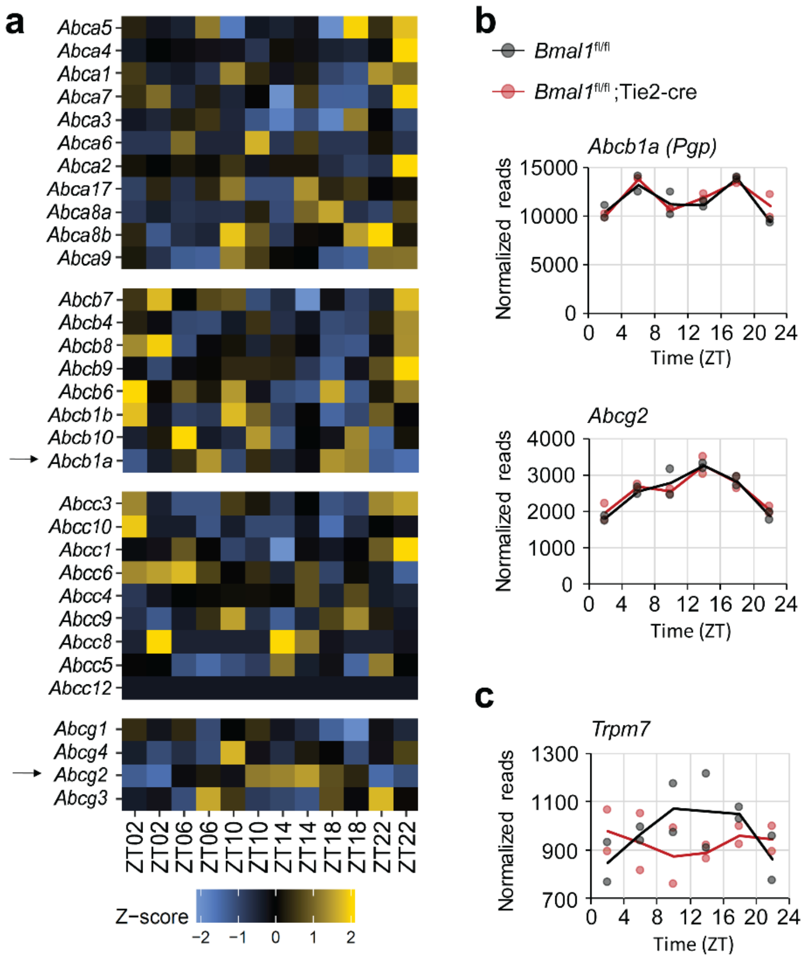

Supplementary Figure 4. Transcripts of ABC family transporters do not display *Bmal1*-dependent rhythms. Transcripts from sorted brain endothelial cells were analyzed using RNA-seq. a) Heatmap of ABC family transporter transcripts of control animals. No ABC family transcripts were found to cycle in control endothelial cells, but not in *Bmal1*-deficient endothelial cells. **b)** Individual plots of *Abcb1* and *Abcg2* show no difference between control and *Bmal1*-deficient endothelial cells. *Abcb1* does not cycle while *Abcg2* is the strongest cycling ABC family transcript in control (black) animals (pCycle <0.001; q < 0.5), but is not different in *Bmal1*-deficient (red) cells. **c)** *Trpm7* transcript levels do not cycle in *Bmal1*-deficient brain endothelium. Individual plot of *Trpm7* in control (black) and *Bmal1*-deficient brain endothelial cells (red) is shown. Controls shows a cyclic, although not significant by JTKCycle (pCycle = 0.1), pattern.

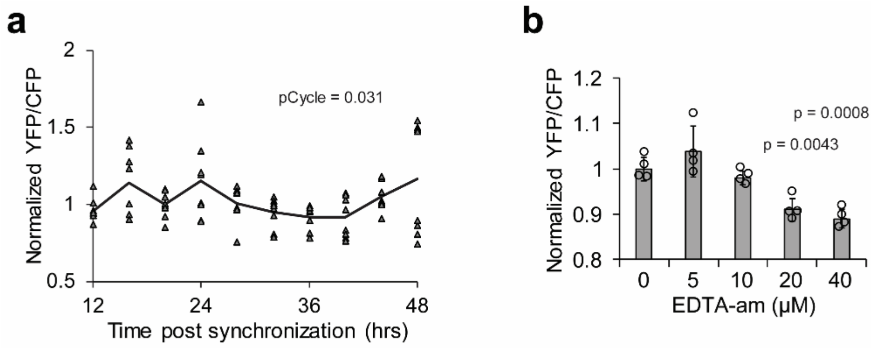

Supplementary Figure 5. Intracellular magnesium levels in hCMEC/D3 cells are cyclic using a genetic FRET sensor. a) Intracellular magnesium levels cycle in hCMEC/D3 cells. hCMEC/D3 cells were made into a stable line expressing MARIO, a genetic FRET sensor for intracellular magnesium. Cells were synchronized with a 30 min pulse of dexamethasone and the cells were measured at ex440/em528 (YFP) and ex440/em480 (CFP) with a plate reader 12 to 48 hours post-synchronization. Data are shown as normalized ratios of YFP to CFP with mean (line) and individual points (triangles). pCycle values were calculated by JTKCycle analysis. n = 40; 2 independent experiments. **b**) Chelating magnesium reduces FRET. Indicated doses of EDTA-AM was added to hCMEC/D3 cells stably expressing MARIO. Fluorescence was measured at ex440/em528 (YFP) and ex440/em480 (CFP). Normalized ratios of YFP to CFP are shown as means ± SEM (n=4; representative of 2 independent experiments). p-values were calculated using one-way ANOVA with Dunnett’s multiple comparisons test.

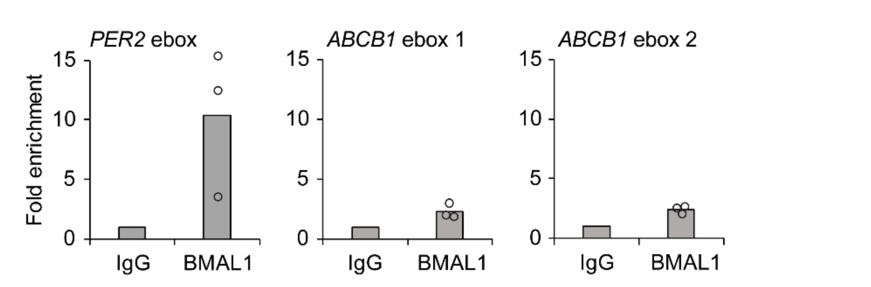

Supplementary Figure 6. Negligible Bmal1 enrichment on ABCB1 ebox sites. Genomic DNA was harvested from hCMEC culture. Chromatin was immunoprecipitated with IgG or Bmal1 antibody and indicated ebox sites were detected by qPCR. *Per2* ebox had approximately 10-fold enrichment of Bmal1 over IgG while ABCB1 ebox sites had 2-fold enrichment. Means and individual points (n=3; 3 independent experiments) are shown.

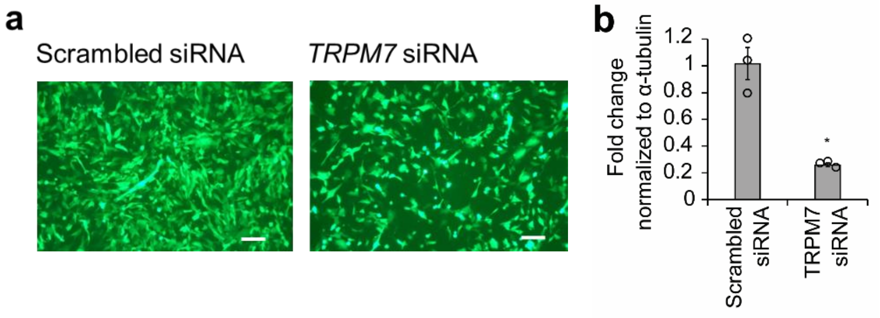

Supplementary Figure 7. Generation of an hCMEC/D3 stable cell line with *TRPM7* knocked down. hCMEC/D3 cells at 70% confluence were infected with lentivirus containing scrambled siRNA with GFP or TRPM7 siRNA with GFP. Viral particles were removed after 24 hrs with media change and cell lines were visually inspected. a) TRPM7 is required for hCMEC/D3 viability. Representative fluorescent images were taken of cell lines 3 days after transduction. TRPM7 siRNA exhibited higher cell death and lower GFP fluorescence compared to controls suggesting higher levels of TRPM7 knockdown reduced cell viability. Scale = 100μm. Data shown are representative of 2 biological replicates. b) TRPM7 siRNA reduced TRPM7 transcript levels to 25%. Cell lines were grown to confluence and assayed for tubulin and *TRPM7* RNA by RT-PCR. Means ± SEM from 3 independent experiments are shown.

Table S4. Primer lists

Real-time PCR primers		
**Mouse transcript**	**Forward primer**	**Reverse primer**
GAPDH	5’-AGGTCGGTGTGAACGGATTTG-3’	5’-TGTAGACCATGTAGTTGAGGTCA-3’
NR1D1	5’-TACATTGGCTCTAGTGGCTCC-3’	5’-CAGTAGGTGATGGTGGGAAGTA-3’
TRPM7	5’-AGGATGTCAGATTTGTCAGCAAC-3’	5’-CCTGGTTAAAGTGTTCACCCAA-3’
**Human Transcript**	**Forward primer**	**Reverse primer**
β-tubulin	5'-TGGACTCTGTTCGCTCAGGT-3'	5'-TGCCTCCTTCCGTACCACAT-3'
TRPM7	5'-GAAGCACCATCTTGGACTCTTGC-3'	5'-GGACCACAGATTTGAGGGATAAC-3'
BMAL1	5'-GCTCAGGAGAACCCAGGTTATC-3'	5'-GCATCTGCTTCCAAGAGGCTCA-3'
ABCB1	5'-ACAACCGGCTTCCGCTTGAGAA-3'	5'-ACGCAGTCGAAAATGAAGCGGC-3'

Primers for targets of Bmal1 chromatin immunoprecipitation		
**Human promoter**	**Forward primer**	**Reverse primer**
hPer2 ebox	5’-ATTGAGGAACCGACGAGGT-3’	5’-GCCCACAGCTGCACGTAT-3’
hABCB1 ebox 1	5’-GCCGCTGTTCGTTTCCTTTA-3’	5’-CAAGAAGCCCTTCTCCCGTG-3’
hABCB1 ebox 2	5’-GGCTTCTTGAGGCGTGGATA-3’	5’-GTCCAGTGCCACTACGGTTT-3’
hTRPM7 ebox	5’-CAACGGAGATACGGCGTGAA-3’	5’-AGCAGAAGCCGAGTCTTTCAT-3’
