# Supplementary material for: A circadian clock regulates efflux by the blood-brain barrier in mice and human cells
Source: Nat Commun. 2021 Jan 27;12:617. doi: 10.1038/s41467-020-20795-9 (PMC7841146; doi:10.1038/s41467-020-20795-9)
Supplement: Supplementary file 5 — Description of Additional Supplementary Files [file 41467_2020_20795_MOESM5_ESM.pdf]

Description of Additional Supplementary Files

Title: Supplementary Dataset 1.

Description: Total read counts of Bmal1wt and Bmal1mut brain endothelial cells (BECs). Brains from control or endothelial-cell Bmal1-deficient mice were collected at ZT2, ZT6, ZT10, ZT14, ZT18, and ZT22 (n=12; 6 time points, 2 independent experiments). BECs were isolated by FACS using CD31 antibody. RNA was extracted and sequenced with HiSeq.

Title: Supplementary Dataset 2.

Description: Cycling genes in Bmal1wt brain endothelial cells (BECs). RNA-sequencing from control BECs collected at ZT2, ZT6, ZT10, ZT14, ZT18, and ZT22 analyzed by Meta2d analysis for 24-hour cycling.

Title: Supplementary Dataset 3.

Description: Cycling genes in Bmal1mut brain endothelial cells (BECs). RNA-sequencing from Bmalfl/fl; Tie2cre BECs collected at ZT2, ZT6, ZT10, ZT14, ZT18, and ZT22 analyzed by Meta2d analysis for 24-hour cycling.
