# Supplementary material for: A circadian clock regulates efflux by the blood-brain barrier in mice and human cells
Source: Nat Commun. 2021 Jan 27;12:617. doi: 10.1038/s41467-020-20795-9 (PMC7841146; doi:10.1038/s41467-020-20795-9)
Supplement: Supplementary file 6 — Reporting Summary [file 41467_2020_20795_MOESM6_ESM.pdf]

nature research

Corresponding author(s): Shirley Zhang, Amita Sehgal

Last updated by author(s): Dec 8, 2020

Reporting Summary

Nature Research wishes to improve the reproducibility of the work that we publish. This form provides structure for consistency and transparency in reporting. For further information on Nature Research policies, see our Editorial Policies and the Editorial Policy Checklist.

Statistics

For all statistical analyses, confirm that the following items are present in the figure legend, table legend, main text, or Methods section.

n/a	Confirmed	
☐	☒	The exact sample size (*n*) for each experimental group/condition, given as a discrete number and unit of measurement
☐	☒	A statement on whether measurements were taken from distinct samples or whether the same sample was measured repeatedly
☐	☒	The statistical test(s) used AND whether they are one- or two-sided<br>*Only common tests should be described solely by name; describe more complex techniques in the Methods section.*
☒	☐	A description of all covariates tested
☒	☐	A description of any assumptions or corrections, such as tests of normality and adjustment for multiple comparisons
☐	☒	A full description of the statistical parameters including central tendency (e.g. means) or other basic estimates (e.g. regression coefficient) AND variation (e.g. standard deviation) or associated estimates of uncertainty (e.g. confidence intervals)
☐	☒	For null hypothesis testing, the test statistic (e.g. *F*, *t*, *r*) with confidence intervals, effect sizes, degrees of freedom and *P* value noted<br>*Give P values as exact values whenever suitable.*
☒	☐	For Bayesian analysis, information on the choice of priors and Markov chain Monte Carlo settings
☒	☐	For hierarchical and complex designs, identification of the appropriate level for tests and full reporting of outcomes
☒	☐	Estimates of effect sizes (e.g. Cohen's *d*, Pearson's *r*), indicating how they were calculated

Our web collection on statistics for biologists contains articles on many of the points above.

Software and code

Policy information about availability of computer code

Data collection	*Provide a description of all commercial, open source and custom code used to collect the data in this study, specifying the version used OR state that no software was used.*
Data analysis	pCycle anaylses were performed with Meta2d or JTKv3.1, which are available from GitHub and have previously published and referenced in the manuscript

For manuscripts utilizing custom algorithms or software that are central to the research but not yet described in published literature, software must be made available to editors and reviewers. We strongly encourage code deposition in a community repository (e.g. GitHub). See the Nature Research guidelines for submitting code & software for further information.

Data

Policy information about availability of data

All manuscripts must include a data availability statement. This statement should provide the following information, where applicable:

- Accession codes, unique identifiers, or web links for publicly available datasets
- A list of figures that have associated raw data
- A description of any restrictions on data availability

The data that support the findings of this study are available upon request. Source data are available for Figures 1b-d, 2, 4c-f, 5c, 6. All relevant data are available from the authors. The RNA-sequencing datasets have been deposited in GEO database under accession code GSE135874 (https://www.ncbi.nlm.nih.gov/geo/query/acc.cgi?acc=GSE135874).

Field-specific reporting

Please select the one below that is the best fit for your research. If you are not sure, read the appropriate sections before making your selection.

- [x] Life sciences
- [ ] Behavioural & social sciences
- [ ] Ecological, evolutionary & environmental sciences

For a reference copy of the document with all sections, see nature.com/documents/nr-reporting-summary-flat.pdf

Life sciences study design

All studies must disclose on these points even when the disclosure is negative.

Sample size	Sample sizes for each experiment was initially determined by previous manuscripts or determined with powerandsamplesize.com
Data exclusions	In cell culture experiments data were excluded if cells in wells were dead/dried up. Exclusion criteria were pre-established.
Replication	Experiments were repeated and most were successful with the exception of experiments using the magnesium FRET sensor. It is likely that the cell line had drifted from selective pressure.
Randomization	Mice were randomly assigned to time points.
Blinding	Surgeon was blinded to the time point and genotype for mouse experiments. Cell culture experiments were not blinded due to the quantitative nature of the experiments.

Reporting for specific materials, systems and methods

We require information from authors about some types of materials, experimental systems and methods used in many studies. Here, indicate whether each material, system or method listed is relevant to your study. If you are not sure if a list item applies to your research, read the appropriate section before selecting a response.

Materials & experimental systems

n/a	Involved in the study	
[ ]	[x]	Antibodies
[ ]	[x]	Eukaryotic cell lines
[x]	[ ]	Palaeontology and archaeology
[ ]	[x]	Animals and other organisms
[x]	[ ]	Human research participants
[x]	[ ]	Clinical data
[x]	[ ]	Dual use research of concern

Methods

n/a	Involved in the study	
[x]	[ ]	ChIP-seq
[ ]	[x]	Flow cytometry
[x]	[ ]	MRI-based neuroimaging

Antibodies

Antibodies used	Depletion antibody: anti-CD90 (clone M5/49.4.1, BioXell); flow cytometry: fluorescence-conjugated anti-CD31 (390, BioLegend), anti-CD90 (53-2.1, Biolegend); ChIP: anti-BMAL1 antibody ab3350, Abcam; WB: anti-PGP (1:1000, MDR-1 c219, ThermoFisher), anti-TRPM7 (1:500, EPR4582, Abcam) anti-BMAL1 (1:1000, A302-616A, Bethyl Laboratories), and anti-beta-Actin (1:20000, mAbcam8224, Abcam).
Validation	All primary antibodies were validated by the manufacturer for species and application OR previously validated (see reference for anti-BMAL1 ab3350)

Eukaryotic cell lines

Policy information about cell lines

Cell line source(s)	Cell line (hCMEC/D3) was sourced by Millapore
Authentication	Cell lines were not authenticated
Mycoplasma contamination	All cell lines tested negative for mycoplasma
Commonly misidentified lines (See ICLAC register)	*Name any commonly misidentified cell lines used in the study and provide a rationale for their use.*

Animals and other organisms

Policy information about studies involving animals; ARRIVE guidelines recommended for reporting animal research

Laboratory animals	Mouse musculus; C57BL/6J, B6.129S4(Cg)-Arntltm1Weit/J, B6.Cg-Tg(Tek-cre)1Y, Cry1-/-Cry2-/-; male and female, 8 weeks - 4 months
Wild animals	*Provide details on animals observed in or captured in the field; report species, sex and age where possible. Describe how animals were caught and transported and what happened to captive animals after the study (if killed, explain why and describe method; if released, say where and when) OR state that the study did not involve wild animals.*
Field-collected samples	*For laboratory work with field-collected samples, describe all relevant parameters such as housing, maintenance, temperature, photoperiod and end-of-experiment protocol OR state that the study did not involve samples collected from the field.*
Ethics oversight	All live animal experiments were performed according to protocols approved by the Institutional Animal Care and Use Committee of the University of Pennsylvania (Philadelphia, PA) in accordance with guidelines set by the NIH

Note that full information on the approval of the study protocol must also be provided in the manuscript.

Flow Cytometry

Plots

Confirm that:

- [x] The axis labels state the marker and fluorochrome used (e.g. CD4-FITC).
- [x] The axis scales are clearly visible. Include numbers along axes only for bottom left plot of group (a 'group' is an analysis of identical markers).
- [x] All plots are contour plots with outliers or pseudocolor plots.
- [x] A numerical value for number of cells or percentage (with statistics) is provided.

Methodology

Sample preparation	Brains were harvested from mice at time points around the circadian day (ZT2, 6, 10, 14, 18, 22).
Instrument	BD FACSAria or BD FACSMelody (BD Biosciences)
Software	Data from sort were analyzed using FlowJo V10.6 software (TreeStar).
Cell population abundance	Between 10000 and 20000 cells were sorted from a single mouse at >95% purity verified by post-sort analysis
Gating strategy	Dead cells were excluded through 4′,6-diamidino-2-phenylindole uptake. Doublets were excluded through FSC-H by FSC-W and SSC-H by SSC-W parameters.

- [x] Tick this box to confirm that a figure exemplifying the gating strategy is provided in the Supplementary Information.
