# Supplementary figures and images for: A circadian clock regulates efflux by the blood-brain barrier in mice and human cells
Source: Nat Commun. 2021 Jan 27;12:617. doi: 10.1038/s41467-020-20795-9 (PMC7841146; doi:10.1038/s41467-020-20795-9)

Fig 5e

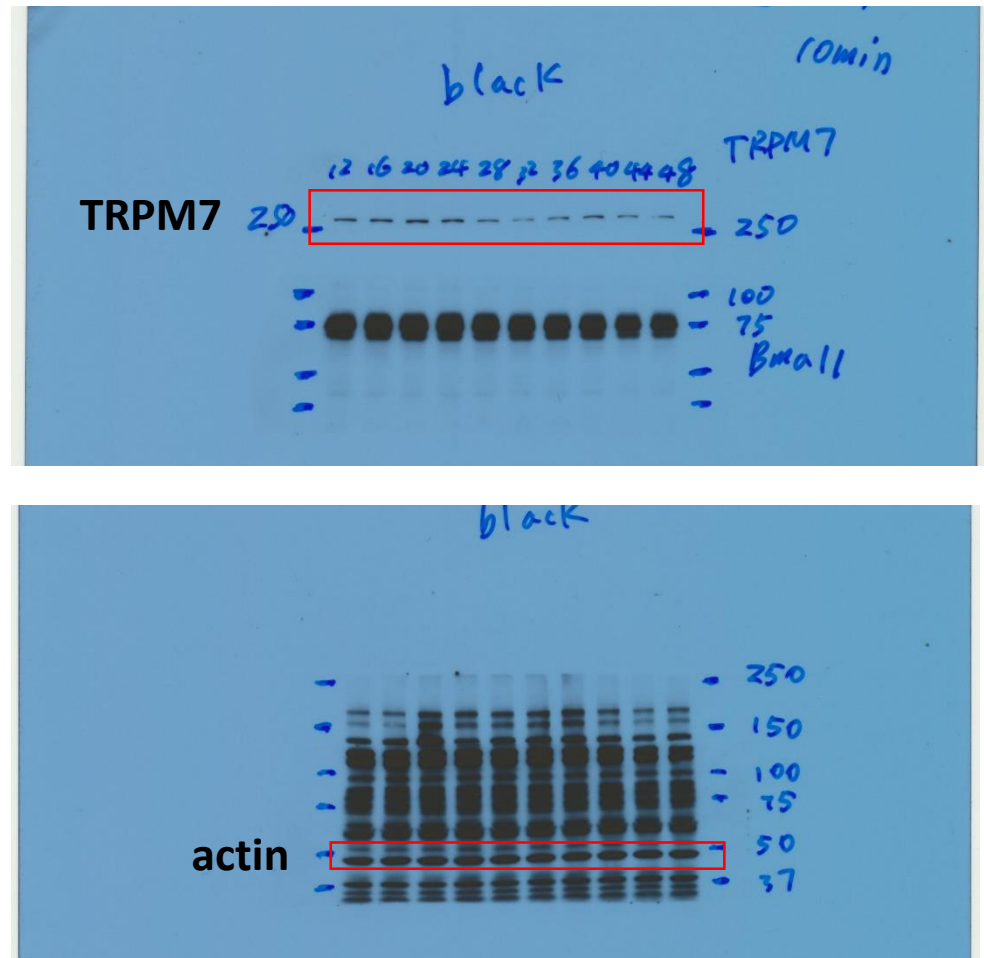

Fig 5f

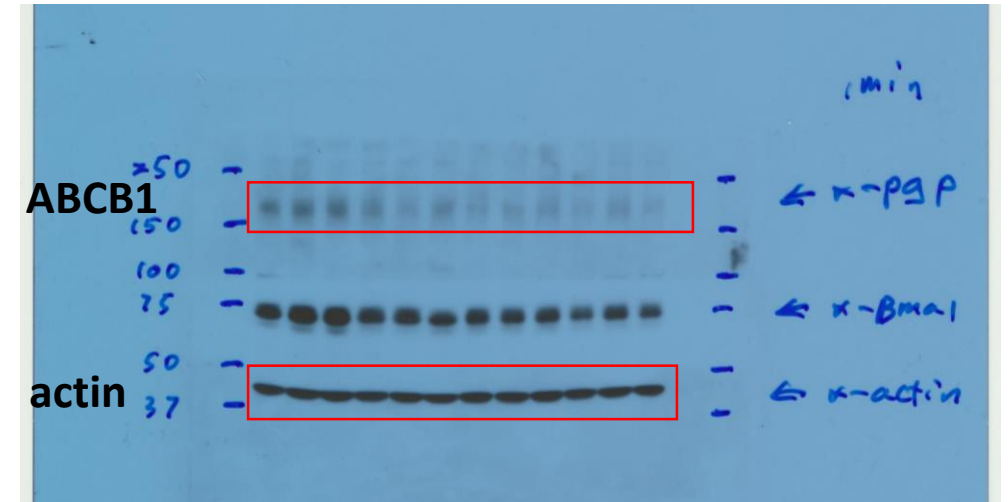

Supplement: Supplementary file 7 — Source Data [file 41467_2020_20795_MOESM7_ESM.zip › WB raw.pdf]
